# Distribution of Virulence Genes and Sequence-Based Types Among *Legionella pneumophila* Isolated From the Water Systems of a Tertiary Care Hospital in India

**DOI:** 10.3389/fpubh.2020.596463

**Published:** 2020-11-23

**Authors:** K. Sreenath, Rama Chaudhry, E. V. Vinayaraj, A. B. Dey, S. K. Kabra, Bhaskar Thakur, Randeep Guleria

**Affiliations:** ^1^Department of Microbiology, All India Institute of Medical Sciences, New Delhi, India; ^2^Geriatric Medicine, All India Institute of Medical Sciences, New Delhi, India; ^3^Pediatrics, All India Institute of Medical Sciences, New Delhi, India; ^4^Biostatistics, All India Institute of Medical Sciences, New Delhi, India; ^5^Pulmonary, Critical Care, and Sleep Medicine, All India Institute of Medical Sciences, New Delhi, India

**Keywords:** *Legionella*, legionellosis, environmental surveillance, virulence, genotyping, sequence-based typing, molecular epidemiology

## Abstract

**Background:** Legionnaires' disease (LD) is a potentially fatal pneumonia predominantly caused by infection due to *Legionella pneumophila* although more than 50 other *Legionella* species are described. Water systems contaminated with *Legionella* spp. are the implicated sources of Legionnaires' disease. In this study, we aimed to assess *Legionella* contamination in the water sources of a tertiary care hospital and to determine the virulence properties and molecular characteristics of *L. pneumophila* environmental isolates.

**Methods:** During May 2015 through August 2018, a total of 201 hospital water samples were tested for *L. pneumophila* by standardized culture procedures; environmental isolates were examined for the presence of two virulence genes: *Legionella vir homolog (lvh)* and *repeats in structural toxin (rtxA)* by PCR. The genotyping of isolates was performed by sequence-based typing (SBT) according to the protocol of the European Study Group for *Legionella* Infections (ESGLI).

**Results:** *L. pneumophila* was isolated from 38/201 (18.9%) water samples; among the 46 isolates, the *lvh* locus was present in 45 (97.8%), the *rtxA* locus was found in 45 (97.8%), and both loci were found in 44 (95.7%) isolates. A total of 23 sequence types (STs) were identified among the 44 isolates (index of discrimination [IOD] of 0.929), and 11/23 (47.8%) STs were new to the ESGLI database.

**Conclusions:** The study results showed genetic diversity in *L. pneumophila* isolates from the hospital environment along with a high percentage of pathogenicity loci. Besides, certain STs may have an increased ability to cause legionellosis, thus requires specific infection control and prevention strategies whenever identified.

## Introduction

*Legionella pneumophila*, the etiological agent of atypical pneumonia known as Legionnaires' disease (LD), is the inhabitants of both natural and human-made aquatic environments (1). LD is the most common waterborne disease, and the reported cases of legionellosis have shown rising trends both in the United States and Europe (2, 3). Presently, the genus *Legionella* includes more than 70 distinct serogroups from >60 known species, and of these, at least 30 species have been associated with opportunistic infections in humans. *L. pneumophila* accounts for ~90% of LD cases, and the majority of clinical cases are attributed to serogroup 1 of *L. pneumophila* (Lp1) (1, 4).

*Legionella* spp. can survive for prolonged periods in aquatic systems, can evade and multiply in free-living protozoa and replicate in the presence of biocides, including chlorine (5). *Legionella* contamination has been increasingly reported in cooling towers (CT), hot springs, foot spas, drinking and non-drinking water systems of hotels, nursing homes, and health care facilities (6, 7). The periodic monitoring of *Legionella* in the hospital water systems allows for risk prediction and the elimination of this pathogen from possible infection sources (7). In India, LD has been sporadically reported from specific locations, but the disease clusters and outbreaks are so far not identified (8, 9). The water safety regulations for *Legionella* monitoring and decontamination are absent in this country, and no active surveillance program exists for monitoring *L. pneumophila* in the hospital environment.

Studies indicate that difference exists between *L. pneumophila* strains, particularly in their ability to withstand in external environments and to produce infections in humans. These differences are mainly attributed to the presence or absence of specific genes encoding virulence among bacterial isolates (10). Several virulence genes in *Legionella* spp. including the type IV secretion system genes, intracellular multiplication/defective in organelle trafficking (*icm/dot*), *tra1, Legionella* vir homolog (*lvh*), type IV pilus genes *pilDE*, macrophage infectivity potentiator (*mip*), repeats in structural toxin (*rtxA*), and *enh*C have been characterized and are extensively studied (10–12). The *lvh* locus derives protein for a second type IV secretion system that contributes to conjugation and virulence (13). The *rtxA* gene is a pore-forming toxin that is regulated by the *dot/icm* complex that contributes to cellular entry and subsequent adherence to the host cell (14). Previous studies have reported that the *lvh* and *rtxA* regions are found more often in strains associated with human disease (10–12). Therefore, in the present study, we aimed to assess *Legionella* contamination in the water systems of a hospital, to identify the *Legionella* species and serogroups involved, and to survey the presence of two pathogenicity loci (the *lvh* and *rtxA*) in environmental isolates to determine their infection potential.

Molecular typing of *L. pneumophila* isolates is foremost important for epidemiological investigation of LD cases, clusters, and outbreaks. An outbreak source can be determined by linking strains from the environment to clinical strains by using different molecular typing methods (15). *L. pneumophila* isolates can be genotyped by sequence-based typing (SBT) using seven loci including five virulence genes (*flaA, pilE, mip, mompS*, and *proA*) and two housekeeping genes (*asd* and *neuA*) as proposed by the European Study Group for *Legionella* Infections (ESGLI) (16–18). The SBT is a rapid, reproducible, and highly discriminatory typing technique and, therefore, widely accepted as a gold standard for LD outbreak investigations and rapid identification of isolates that are closely related (4, 16, 17). Previous typing studies on *L. pneumophila* isolates indicate the dominance of certain sequence types (STs) in sporadic cases and outbreaks (4). However, from India, so far, no studies were reported to determine the DNA SBT of *L. pneumophila* isolates. Therefore, we further characterized *L. pneumophila* environmental isolates by SBT analysis and compared our data with the global database available at the ESGLI.

## Materials and Methods

### Environmental Surveillance

The study was conducted in a major tertiary care hospital in New Delhi, India, that has organ transplantation and cancer treatment facilities. During May 2015 through August 2018, 21 sites inside the hospital campus that spread over ~115 acres, including hospital, residential, and general areas, were monitored for the presence of *L. pneumophila*. Samplings were performed in different buildings hosting patient rooms, intensive care units, clinics, laboratories, and nursing stations. Both potable (PW; drinking water for patients, hospital staff, and public) and non-potable water samples (NPW; bathwater and water for handwashing) were collected, and *Legionella* testing was carried out four times per year. The samples were collected from distal outlets of hot and cold water taps and AC cooling towers (basin beneath the tower). The water temperature was measured at the time of sample collection by using a precision thermometer (Zeal, England). *Legionella* isolation and identification from water samples was done following the guidelines issued by the US Centers for Disease Control and Prevention (19). The detailed methodology regarding *L. pneumophila* environmental surveillance has been published elsewhere (20, 21).

### *Legionella* Speciation and Identification of *L. pneumophila* Serogroup 1

All *Legionella* isolates collected during the surveillance period were subjected to a real-time PCR assay targeting the *ssrA* gene for the confirmation of the *Legionella* genus (22). Further identification of *L. pneumophila* was done by using a real-time PCR targeting the *mip* gene, and finally, the detection of Lp1 was done by using another real-time PCR targeting *wzm* gene (22). DNA isolated from *L. pneumophila* serogroup 1 ATCC 33152 (Strain Philadelphia) was used as a positive control for the standardization of real-time PCR.

### Identification of Virulence Genes by Using PCR

All *L. pneumophila* isolates recovered from hospital water samples were surveyed for the presence of two virulence gene loci using previously published primers and PCR conditions (10, 11). Briefly, six-primer pairs were used in this study including lvh1/*prpA*-lvh2/*prpA*, lvh3/*lvhB3*-lvh4/*lvhB4*, lvh5/*lvhB8*-lvh6/*lvhB9*, and lvr1/*lvrE*-lvr2/*lvrE* for the amplification of the *lvh* region and rtx1/*rtxA*-rtx2/*rtxA* and rtx3/*rtxA*-rtx4/*rtxA* for the identification of the *rtxA* region. DNA extraction was performed by emulsifying 2–3 colonies in sterile water and boiling at 100°C for 10 min. PCR amplification involved 35 cycles of 1 min at 95°C for denaturation, 1 min at 55°C for annealing, and 1 min at 72°C for the extension. DNA isolated from *L. pneumophila* serogroup 1 ATCC 33152 (Strain Philadelphia) was used as a positive control for PCR.

### *L. pneumophila* Genotyping by Sequence-Based Typing (SBT)

*L. pneumophila* SBT was performed by using the seven-gene (*flaA, pilE, asd, mip, mompS, proA, neuA)* protocol SBT scheme according to the guidelines issued by the ESGLI (version 5.0) (16–18). PCR amplification involved 35 cycles of 30 s at 94°C for denaturation, 30 s at 55°C for annealing, and 45 s at 72°C for the extension. After purification and sequencing (Barcode Biosciences Pvt. Ltd., Bangalore, India; Dr. KPC Life Sciences Pvt. Ltd., Kolkata, India), the forward and reverse sequence trace files were uploaded to the online *Legionella* Sequence Quality Tool (www.hpa-bioinformatics.org.uk/cgi-bin/legionella/sbt/seq_assemble_legionella1.cgi). Sequence alignment and trimming was performed by the tool and individual alleles, allelic profile, and a sequence type (ST) were identified. For each isolate, the profile of seven alleles at each of the loci was defined in the following order: *flaA, pilE, asd, mip, mompS, proA*, and *neuA* (e.g., 1, 4, 3, 1, 1, 1, 1). Finally, the ST was indicated by a number (e.g., ST1). For *L. pneumophila* non-Lp1 strains, if *neuA* is not amplified with standard *neuA* primers, amplification was done by using primers targeting *neuAh* (N-Acylneuraminate Cytidyltransferase homolog) according to an alternative ESGLI protocol (version 1.0) (23). The homolog (*neuAh*) has been described by Farhat et al. and is reported to be found in certain non-serogroup 1 (non-Lp1) strains when the *neuA* gene is not amplified with the standard *neuA* primers in the SBT protocol (23). Therefore, whenever the *neuA* gene is not amplified, in place of *neuA* result, the *neuAh* allele result was used according to the predetermined SBT order (*flaA, pilE, asd, mip, mompS, proA*, and *neuAh)* as recommended by the ESGLI (version 1.0). Finally, the combination of alleles is defined as 7-digit allelic profile (e.g., 8, 6, 34, 9, 2, 8, 209) and a ST was represented by a number (e.g., ST1417). Newly identified alleles and STs were submitted to the ESGLI SBT database (http://www.hpa-bioinformatics.org.uk/legionella/legionella_sbt/php/sbt_homepage.php).

### Phylogenetic Analysis

We assessed the relationships between STs and within clonal complexes by using the goeBURST implemented in the PHYLOViZ program (http://www.phyloviz.net/goeburst/#Software). The default setting in the eBURST (the stringent group definition) was used; by this definition, a clonal complex contains STs that share six of the seven evaluated SBT alleles with at least one other member of the group and are all believed to be descended from the same founding genotype (primary founder) (24). Comparative goeBURST analysis was used to relate *L. pneumophila* STs identified in this study with those reported from Japan, China, and South Korea. The SBT data of 164 isolates from China, 135 isolates from Japan, and 104 isolates from South Korea were obtained from previous studies (6, 25, 26).

### Statistical Analysis

Categorical variables were expressed in terms of numbers and percentages. The association between characteristics of water samples and *L. pneumophila* positivity was evaluated through odds ratio (OR) with 95% CI (confidence interval). A *p*-value (two-tailed) below 0.05 was considered to be statistically significant. The analysis was performed by using function odds. ratio in R-version 3.6.1. The index of diversity (IOD) was determined using Hunter and Gaston's modification of Simpson's index of diversity according to a previously described method (27).

## Results

### Environmental Surveillance

Among the 201 water samples collected during the study period, 38 (18.9%; 19 potable and 19 non-potable) were positive for *L. pneumophila* by culture. Of these, 10 (26.3%) each sample was collected from patient areas, residential areas, and general hospital areas, and 8 (21%) were from AC cooling towers. Regarding the 38 samples tested positive for *L. pneumophila*, the presence of Lp1 was detected in 25 (65.8%) samples, which included 11 potable and 14 non-potable water samples. The remaining 13 (34.2%) samples were contaminated due to Lp 2-14 serogroups. The temperature of *L. pneumophila* positive water samples ranged from 12 to 57°C (median temperature of 25°C), and the pathogen was most frequently isolated from a temperature range of 20°C-40°C (during 29 instances).

Among the 21 sampling sites, 15 were positive for *L. pneumophila* during at least one sampling event, two sites were positive for *Legionella* during two instances, three sites were positive during three instances, and four sites were positive during >4 instances. These four sites (two drinking water units and two AC cooling towers) that repeatedly tested positive for *L. pneumophila* were identified as high-risk sites. *L. pneumophila* positivity for the sampling sites and buildings located within these sites are shown in Table 1.

**Table 1:** *Legionella pneumophila* positivity according to water sampling sites and buildings in a Tertiary care hospital, India, 2015–2018.

**Site ID**	**Hospital buildings located in the site**	**Sampling events**	***L. pneumophila* positive instances**	**Lp1**	**Lp 2-14**
1	Outpatient department (OPD)	6	1	1	–
2	Emergency	4	–	–	–
3	Hospital wards (floor 1–4)	6	–	–	–
4	Hospital wards (floor 5–8)	5	–	–	–
5	Ophthalmology	9	2	2	–
6	Oncology	12	1	1	–
7	Cardiothoracic and neurology	11	3	1	2
8	Pulmonology and new private ward	10	3	3	–
9	Nursing college, genetic lab, community medicine	15	6	5	1
10	Dentistry	5	–	–	–
11	AC cooling tower for OPD building	7	4	3	1
12	Teaching divisions	15	2	2	–
13	General areas near teaching block and library	5	–	–	–
14	Biotechnology	10	1	1	–
15	AC cooling towers for oncology, cardiology, and neurology buildings	26	4	2	2
16	Girls hostels	7	1	1	–
17	Boys hostels	11	3	2	1
18	Swimming pool	2	–	–	–
19	Guest house and staff houses	15	5	–	5
20	New Resident doctors hostels	11	1	–	1
21	Other general areas and laboratories	9	1	1	–

*Lp, Legionella pneumophila*.

We compared water samples with and without *L. pneumophila* and assessed characteristics including type, source, and temperature range of water samples, and age of water tanks or storage systems. None of these factors were found to have a significant association with *L. pneumophila* positivity (Table 2). During the study period, seasonal variations in *Legionella* positivity were not observed; the contamination was found to be consistent throughout the year.

**Table 2:** *Legionella pneumophila* isolated from 201 water samples collected at a tertiary healthcare center, by type of water, sampling site, temperature, year and period, India, May 2015– August 2018.

**S.no**	**Characteristic**	**Total no of samples collected (*n* = 201)**	**Lp positive (*n* = 38) [n (%)]**	**Lp negative (*n* = 163) [n (%)]**	**OR 95%CI**	***p*-value**
1	**Type of water samples**					
	a. Potable b. Non-potable	113 88	19 (16.8) 19 (21.5)	94 (83.2) 69 (78.4)	1.00 1.36 (0.63–2.94)	0.391
2	**Sampling site**					
	a. Patient areas b. Residential areas c. Cooling towers d. Other areas	68 44 33 56	10 (14.7) 10 (22.7) 8 (24.2) 10 (17.9)	58 (85.3) 34 (77.3) 25 (75.8) 46 (82.1)	1.00 1.71 (0.57–5.01) 1.86 (0.56–5.91) 1.26 (0.43–3.69)	0.279 0.240 0.635
3	**Temperature range**					
	a. <20°C b. 20–29°C c. 30–39°C d >40°C	39 118 40 4	8 (20.5) 18 (15.3) 11 (27.5) 1 (25)	31(79.5) 100 (84.7) 29 (72.5) 3(75)	1.00 0.70 (0.26–2.04) 1.47 (0.46–4.84) 1.29 (0.02–18.69)	0.444 0.467 0.834
4	**Seasonality**					
	a. Winter (Dec-Jan) b. Spring (Feb-March) c. Summer (April-June) d. Monsoon (July- August) e. Autumn (Sept-Nov)	20 23 60 44 54	4 (20) 3 (13) 13 (21.7) 8 (18.2) 10 (18.5)	16 (80) 20 (86.9) 47 (78.3) 36 (81.8) 44 (81.5)	1.00 0.60 (0.08–4.17) 1.11 (0.28–5.32) 0.89 (0.20–4.64) 0.91 (0.22–4.54)	0.538 0.875 0.863 0.885
5	**Periodicity**					
	a. First period (Feb, 2015-Jan, 2017) b. Second period (Feb, 2017-Sept, 2018)	79 122	21 (26.6) 17 (13.9)	58 (73.4) 105 (86.1)	1.00 0.45 (0.20–0.97)	**0.030**
6	**Age of building**					
	a. >10 years b. <10 years	187 14	36 (19.3) 2 (14.3)	151(80.7) 12 (85.7)	1.00 0.70 (0.07 – 3.36)	0.647

*CI, confidence interval; Lp, Legionella pneumophila; OR, odds ratio. Bold text indicates statistical significance*.

### *Legionella* Speciation and Identification of *L. pneumophila* Serogroup 1

A total of 47 *L. pneumophila* isolates were obtained from all positive samples during the study period. The number of *Legionella* isolates is not equal to the number of positive samples because, during some instances, we isolated different *L. pneumophila* serogroups (both Lp1 and Lp 2-14 [non-Lp1]) in the same sample. All isolates (*n* = 47) were identified as *L. pneumophila* by real-time PCR targeting the *mip* gene. Twenty-nine out of 47 (61.7%) isolates were identified as Lp1 by *wzm* gene real-time PCR, and the remaining isolates (*n* = 18 [38.3%]) were referred to as Lp 2-14. Serogrouping for the non-Lp1 isolates was not performed.

### Identification of Virulence Genes by Using PCR

**Figure 1:**
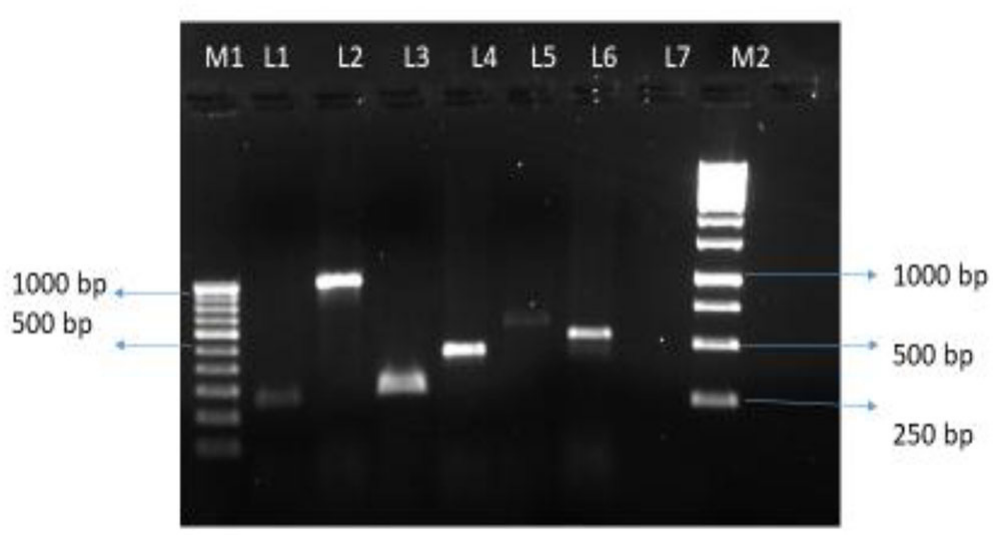
*Legionella pneumophila* isolate from water tested positive for six pairs of primers. Lane M1: 100 bp DNA ladder (100–1,000 bp) with DNA sizes indicated. Lane 1 to 6, PCR products for primer pairs for the *lvh* region: lvh1/*prpA*-lvh2/*prpA* (L1, 259 bp PCR product), lvh3/*lvhB3*-lvh4/*lvhB4* (L2, 1007 bp PCR product), lvh5/*lvhB8*- lvh6/*lvhB9* (L3, 294 bp PCR product), and lvr1/*lvrE*-lvr2/*lvrE* (L4, 423 bp PCR product), and for the *rtxA* region: rtx1/*rtxA*-rtx2/ *rtxA* (L5, 603 bp PCR product), and rtx3/*rtxA*-rtx4/*rtxA* (L6, 543 bp PCR product), Lane 7, blank, Lane M2: 1 Kb DNA ladder with representative sizes of DNA are indicated.

Of 47 *L. pneumophila* isolates, 46 were subjected to the identification of virulence genes. For one isolate, DNA was found to be degraded; therefore, excluded from the analysis. Among the tested isolates, at least one virulence gene loci (*lvh* or *rtxA*) was detected in all (100%) isolates. Specifically, the *lvh* locus was present in 45 (97.8%) isolates, the *rtxA* locus was found in 45 (97.8%), and both loci were found in 44 (95.7%) isolates. The simultaneous absence of the two loci was not observed in any of the tested isolates. Among the two groups (Lp1 and non-Lp1), both the gene loci were present in all Lp1 (*n* = 29, 100%) isolates tested. Of the non-Lp1 (*n* = 17) isolates, 15 (88.3%) tested positive for both genes, and the remaining two isolates showed the following pattern: *lvh*-positive, *rtxA-*negative and *lvh-*negative, *rtxA-*positive. Figure 1 shows PCR positive products in an environmental isolate tested for all primer pairs. Test results of the detection of virulence genes are shown in Table 3.

**Table 3:** Detection of virulence genes in *Legionella pneumophila* isolates from water samples.

***Legionella* spp**.	**No of isolates tested**	***lvh* locus n (%)**	***rtxA* locus n (%)**	***lvh* and *rtxA* loci n (%)**
*L. pneumophila* serogroup 1(Lp1)	29	29 (100)	29 (100)	29 (100)
*L. pneumophila* serogroup 2-14 (non-Lp1)	17	16 (94.1)	16 (94.1)	15 (88.2)
Total	46	45 (97.8)	45 (97.8)	44 (95.6)

### *L. pneumophila* Genotyping by Sequence-Based Typing (SBT)

SBT analysis assigned 44 environmental isolates into 23 distinct STs (IOD, 0.929). DNA of three isolates was found to be degraded; hence genotyping was not applied. For one Lp1 isolate, ST was not able to determine (Table 4). Six STs were associated with more than one isolate, and 17 STs were identified with one single isolate. The most predominant ST in this study was ST1, with 22.7% (10/44) of the isolates belonging to this sequence type. The other STs obtained more than once among the tested isolates were ST763 (*n* = 4; 9.1%), ST2848 (*n* = 4; 9.1%), ST2854 (*n* = 3; 6.8%), ST2868 (*n* = 3; 6.8%), and ST114 (*n* = 2; 4.5%). We identified the presence of the *neuAh* allele in 8 non-Lp1 isolates, which were not amplified using the standard *neuA* primers. They contained five different *neuAh* alleles, including *neuAh* 201, 207, 208, 209, and 228 (Table 4).

**Table 4:** Result of molecular typing of *L. pneumophila* isolates by sequence-based typing (SBT).

**S. no**	**Strain**	**Year of isolation**	**Source**	Allelic profile (flaA,pile,asd,mip,momps,proA,neuA/neuAh^***^)	**sequence Type (ST)**	**SG**
1	AIIMSLP001	2018	NP	1,4,3,1,1,1,1	ST1	1
2	AIIMSLP002	2018	CT	1,4,3,1,1,1,1	ST1	1
3	AIIMSLP003	2018	P	6,10,19,28,19,4,11	ST763	1
4	AIIMSLP004	2018	P	3,10,1,28,14,9,13	ST93	1
5	AIIMSLP005	2018	P	1,4,3,1,1,1,1	ST1	1
6	AIIMSLP006	2018	P	1,4,3,1,1,1,1	ST1	1
7	AIIMSLP007	2018	P	1,4,3,10,2,30,1	ST2848*	1
8	AIIMSLP008	2018	NP	6,10,19,3,19,4,3	ST322	1
9	AIIMSLP009	2018	NP	8,6,3,8,2,8,56(−1)^**^	ND	1
10	AIIMSLP010	2018	P	1,4,3,1,1,1,1	ST1	1
11	AIIMSLP011	2018	NP	1,4,3,1,1,1,1	ST1	1
12	AIIMSLP012	2018	P	1,4,3,1,1,1,1	ST1	1
13	AIIMSLP013	2015	NP	2,10,20,93^*^,21,4,207^***^	ST2854^*^	2-14
14	AIIMSLP014	2015	CT	2,10,20,93^*^,21,4,207^***^	ST2854^*^	2-14
15	AIIMSLP015	2015	NP	1,4,3,1,93,30,1	ST2210	1
16	AIIMSLP016	2015	NP	1,4,3,10,1,1,1	ST134	1
17	AIIMSLP017	2015	CT	1,4,3,10,2,30,1	ST2848^*^	1
18	AIIMSLP018	2015	P	2,10,15,28,19,4,3	ST1464	1
19	AIIMSLP019	2015	P	5,2,22,27,6,10,12	ST48	1
20	AIIMSLP020	2015	P	1,4,3,1,1,1,1	ST1	1
21	AIIMSLP021	2015	CT	1,4,3,10,2,30,1	ST2848^*^	1
22	AIIMSLP022	2015	NP	7,4,3,10,1,1,13	ST2869^*^	1
23	AIIMSLP023	2016	NP	1,4,3,1,1,1,1	ST1	1
24	AIIMSLP024	2016	P	6,10,19,28,19,4,11	ST763	1
25	AIIMSLP025	2016	P	1,4,3,1,1,1,1	ST1	1
26	AIIMSLP026	2016	P	6,10,19,28,19,4,11	ST763	1
27	AIIMSLP027	2015	CT	6,10,19,28,19,4,11	ST763	1
28	AIIMSLP028	2016	P	3,6,1,6,14,11,9	ST114	1
29	AIIMSLP029	2018	CT	1,4,3,10,2,30,1	ST2848^*^	1
30	AIIMSLP030	2016	P	3,6,1,6,14,11,9	ST114	2-14
31	AIIMSLP031	2016	P	8,6,34,9,2,8,209^***^	ST1417	2-14
32	AIIMSLP032	2015	NP	6,10,15,28,21,14,11	ST1095	2-14
33	AIIMSLP033	2018	P	1,4,3,1,1,30,6	ST2849^*^	2-14
34	AIIMSLP034	2015	P	3,10,1,5,14,9,3	ST2868^*^	2-14
35	AIIMSLP035	2015	CT	2,10,20,93^*^,21,4,207^***^	ST2854^*^	2-14
36	AIIMSLP037	2015	NP	2,10,3,28,19,4,3	ST2850^*^	2-14
37	AIIMSLP038	2018	P	1,4,3,16,2,1,208^***^	ST1376	2-14
38	AIIMSLP039	2016	P	6,4,19,28,19,4,15	ST2855^*^	2-14
39	AIIMSLP040	2018	P	2,10,15,12,19,4,3	ST2867^*^	2-14
40	AIIMSLP041	2018	CT	6,10,15,14,21,4,207^***^	ST2866^*^	2-14
41	AIIMSLP043	2016	P	3,10,1,5,14,9,3	ST2868^*^	2-14
42	AIIMSLP044	2015	CT	8,49,34,8,12,8,228^***^	ST2865^*^	2-14
43	AIIMSLP045	2015	P	3,10,1,5,14,9,3	ST2868^*^	2-14
44	AIIMSLP046	2016	P	3,10,1,28,1,9,201^***^	ST2874^*^	2-14

*CT, cooling tower; NP, non-potable water; ND, not determined; P, potable water; SBT, sequence-based typing; SG serogroup*.

* *Newly identified alleles and sequence types (STs) in the present study*.

** *The ST of this isolate was not able to determine; neuA sequence results showed the closest match to neuA 56*.

*** *The non-Lp1 isolates tested positive for the neuAh allele. The neuAh nomenclature starts from 201, to differentiate between the neuA and neuAh alleles. Bold text indicates newly identified allele and STs in this study*.

Querying the ESGLI database (available at http://www.ewgli.org), it was found that 11/23 (47.8%) STs were identified for the first time in this study. They are ST2848, ST2849, ST2850, ST2854, ST2855, ST2865, ST2866, ST2867, ST2868, ST2869, and ST2874. We also identified one new allele of the *mip* gene (*mip* 93) (Table 4).

Strains with indigenous STs were isolated from different water sources. Nine cooling tower (CT) isolates were divided into 6 STs (IOD, 0.888), 24 PW isolates were divided into 14 STs (IOD, 0.920), and 11 NPW isolates were divided into 8 STs (IOD, 0.933; except one isolate of which ST was not determined). ST1 was found in all three sources (PW; *n* = 6, NPW; *n* = 3, and CT; *n* = 1). Other common STs found across different water environments included ST2854 (CT; *n* = 2, NPW; *n* = 1), ST2848 (CT; *n* = 3, PW; *n* = 1), and ST763 (PW; *n* = 3, CT; *n* = 1). The only ST common across the patient, residential, and general areas of this hospital was ST1. Apart from ST1, 5 STs including ST114, ST322, ST1095, ST2849 (a new ST), and ST2869 (a new ST) were found to be present in the patient areas. Twenty-seven isolates of Lp1 were assigned into 11 STs (IOD, 0.824; except one isolate of which ST was not determined), and 17 non-LP1 isolates were assigned into 14 STs (IOD, 0.955). The common ST appeared in both groups (Lp1 and non-Lp1) was only ST114.

### goeBURST Analysis

**Figure 2:**
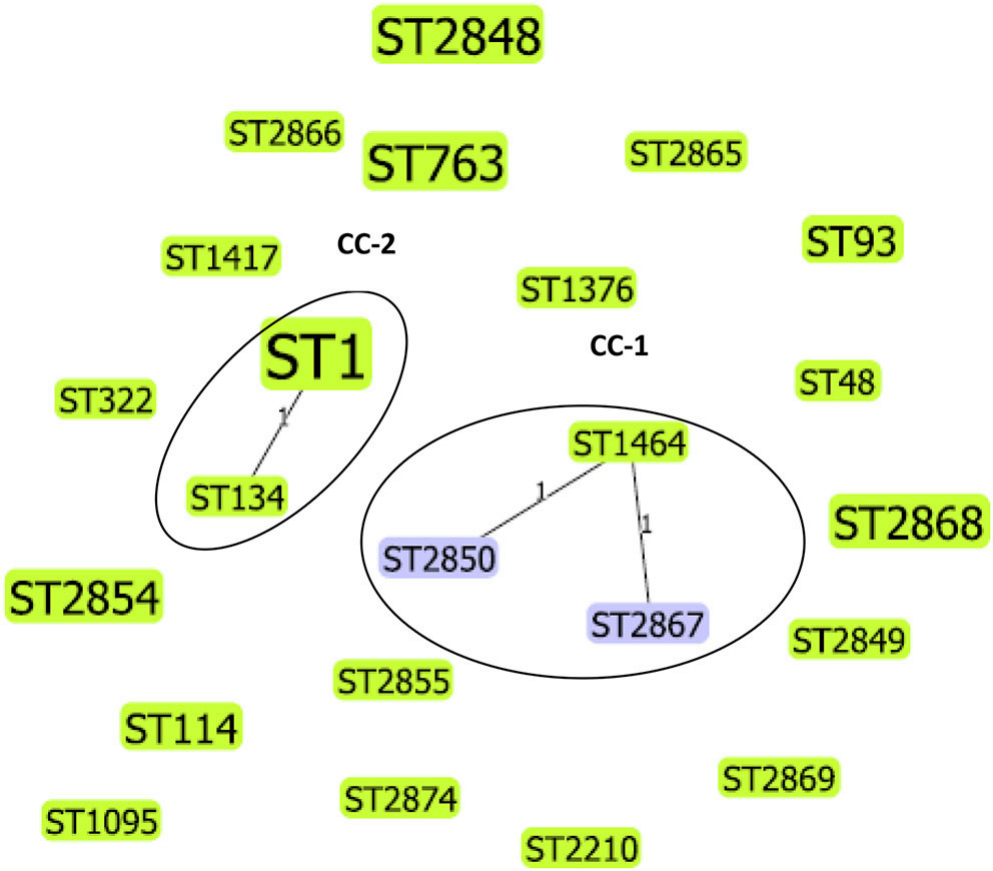
Phylogenetic relationship between *L. pneumophila* STs identified by goeBURST analysis, only the single-locus variants (SLV), is shown as a population snapshot. goeBURST analysis of 23 STs showed that 5 of these STs were predicted to form 2 clonal complexes, and 18 STs did not relate to each other and existed as singletons. Clonal complex 1 (CC-1) consisted of three STs and 3/44 (6.82%) isolates. Clonal complex 2 (CC-2) consisted of 2 STs and 11/44 (25%) isolates. The size of the ST node in the figure reflects the abundance of that ST in the input data. The determined group founder is shown in light green, and common nodes are shown in blue.

We applied goeBURST analysis to examine the relationship between STs obtained in this study with a single-locus variant selected (SLV). Five STs from this study were predicted to form two clonal complexes, whereas the remaining 18 STs did not relate to each other, therefore, identified as singletons. Clonal complex 1 (CC-1) consisted of 3 STs (ST1464, ST2850, ST2867), and 3/44 (6.8%) isolates belonged to this complex. The CC-2 consisted of two STs (ST1 and ST134), and 11/44 (25%) isolates belonged to this complex. The phylogenetic relationship between *L. pneumophila* STs identified by goeBURST analysis is shown in Figure 2.

### Comparative goeBURST Analysis of *L. pneumophila* Environmental Isolates From India, China, Japan, and South Korea

**Figure 3:**
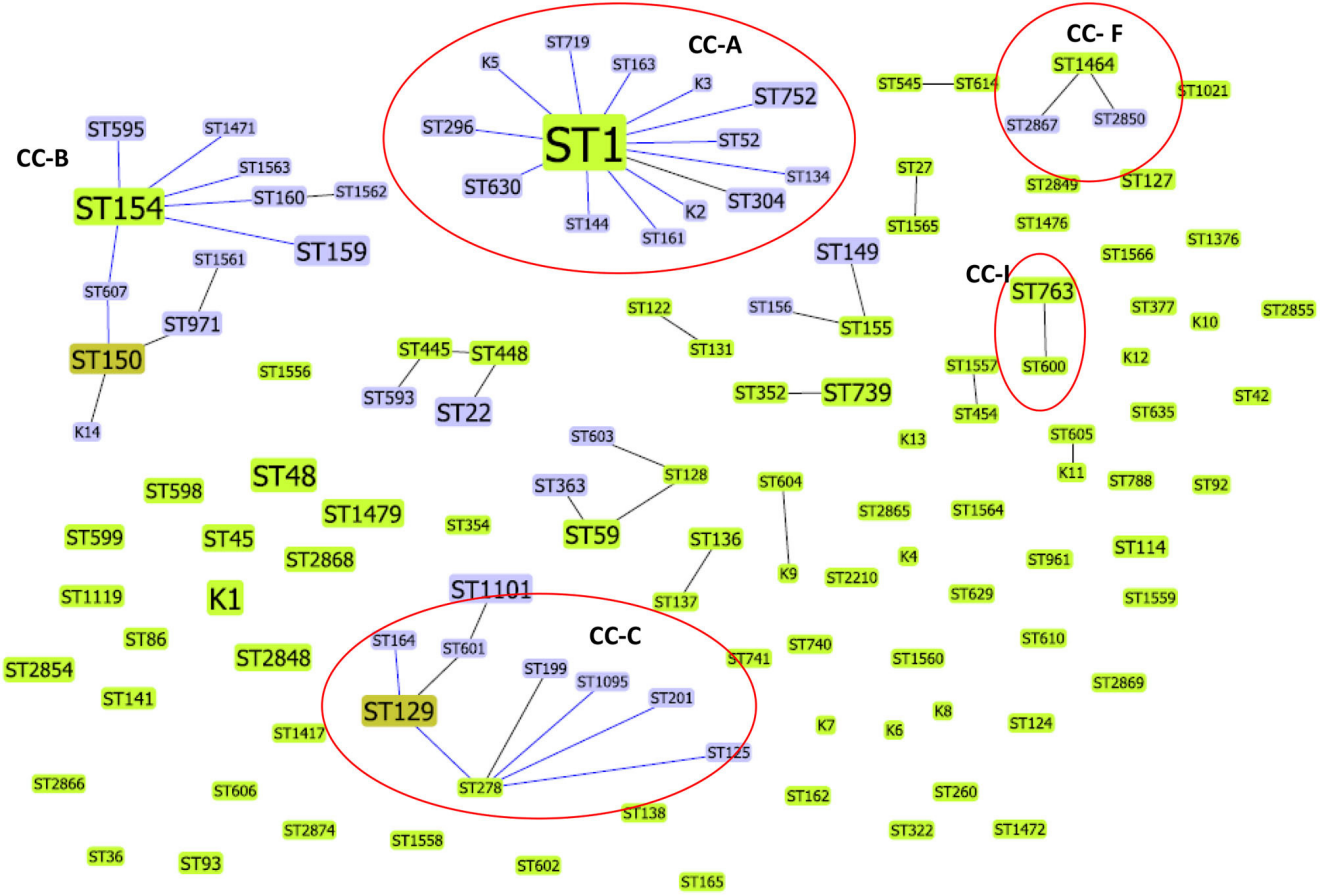
Comparative goeBURST analysis of *L. pneumophila* STs detected in this study with environmental *L. pneumophila* serogroup1 STs reported from China, Japan, and South Korea. Only single-locus variant (SLV) links are shown. The population snapshot contained 16 clonal complexes and 60 singletons. The group founders are shown in light green ST nodes, and dark green ST nodes represent the sub-group founders. Blue ST nodes are the common nodes. The clonal complexes CC-A, CC-C, CC-F, and CC-I (shown in circles) contained STs (ST1, ST134, ST763, ST1095, ST1464, ST2850, ST2867) identified in this study.

Forty-four *L. pneumophila* isolates detected in the present study were compared with 403 Lp1 environmental isolates from Japan (*n* = 135), China (*n* = 164), and South Korea (*n* = 104). The SBT data of Chinese, Japanese, and South Korean isolates were obtained from previously published studies (6, 25, 26). Altogether, these 447 isolates were divided into 127 STs. Most of these STs exclusively belonged to one country, and the only ST that found to be present in all the four countries was ST1. Apart from ST1, ST1464 detected in this study was also reported from China; similarly, ST48 was also reported from Japan. The comparative goeBURST analysis grouped 67 STs into 16 clonal complexes (CCs), and 60 STs were identified as singletons (Figure 3). Among the 16 CCs, four clonal complexes (CC-A, CC-C, CC-F, and CC-I) also contained STs identified in this present study.

ST1 was the determined primary founder of the largest clonal complex CC-A; it had 13 SLVs, including ST134, which is detected in the present study. The CC-C with determined primary founder ST278 and subgroup founder ST129 contained ST1095, a ST identified in our study that shares 6/7 alleles with ST278 (ST reported from Japan). The CC-F, which is observed in the present study, contained ST1464 as a predicted primary founder and ST2867 and ST2850 as obtained SLVs. Another ST reported in this study, ST763 belonged to CC-I and is closely related to ST600 (a ST reported from Japan) (Figure 3).

## Discussion

### Environmental Surveillance

Drinking water colonization by *Legionella* spp. is directly linked to the occurrence of HALD, and several national public health agencies have mandated routine environmental surveillance as a preventive measure. In our study, *L. pneumophila* was isolated from 18% of hospital water samples, and these findings are in line with a previous study from India that reported a positivity of 15% (28). In studies reported from Spain and Italy, the occurrence of *Legionella* in the hospital environment has been found to be higher (60 and 74.1%, respectively) (29, 30). Surveys of *Legionella* colonization in hospitals have been performed in the USA, UK, Canada, and Spain, with *Legionella* positivity rates varied from 12 to 85% (31, 32). In a large-scale hospital survey conducted in Taiwan (belonging to East Asia), *L. pneumophila* contamination was found in the water systems of 10 out of 16 hospitals (33).

In the present study, during 19 instances, potable water tested positive for *L. pneumophila*, and it is reported that in hospital settings, potable water rather than cooling towers has been implicated as a potential source of legionellosis (7). One possible solution to prevent the spread of *Legionella* from the water was the application of filters to the taps, thus allowing safe water free of the pathogen (34). Therefore, as an initial infection control measure, point-of-use filters were applied to the drinking water taps from where *Legionella* was isolated. Repeat sampling from these sites did not show *Legionella* re-colonization after 6 and 12 months. However, to evaluate the efficacy of any *Legionella*-disinfection method, monitoring over a prolonged period is required. The cooling towers were cleaned and disinfected at least once every 12 months, and water was sampled and tested at least once every 3 months for *Legionella* spp. These sites were found to be positive for *Legionella* after 4 months, and the bacteria were isolated from cooling towers until they closed for the next cycle of annual maintenance. *Legionella* isolation rates reduced significantly in water systems from an average of 26.6% during Feb 2015- January 2017 to an average of 13.9% during February 2017-September 2018 (OR 0.45; 95% CI 0.20–0.97; *p* = 0.03). This reduction in *Legionella* isolation rates could be due to *Legionella*-specific interventions and control measures.

As a part of *Legionella* risk management, the physicians were informed regarding *Legionella* colonization in the hospital water systems, and intensive clinical surveillance for this pathogen was initiated. Furthermore, in the future, if a high-level *Legionella* colonization is observed in this facility, it is pertinent to install a systemic disinfection system with long term-commitment with a specific aim of preventing legionellosis in the exposed population.

### Detection of Virulence Genes

The *lvh* and *rtxA* loci are seen frequently in *L. pneumophila* isolates associated with human infections. Therefore, these loci can be used as markers for determining the infection potential of isolates (10–12). Our results showed that these genetic loci are found at a very high percentage in *L. pneumophila* strains from hospital water systems. This finding is in agreement with studies from Greece, Australia, and China that reported a high percentage of the pathogenicity loci in *L. pneumophila* environmental isolates (11, 35, 36). Despite the high prevalence *L. pneumophila* containing virulence genes in our hospital environment, HALD clusters were not identified during the clinical surveillance. Similarly, even though *Legionella* colonization was observed in 24% of tap waters in Singapore, HALD cases were not identified during 1998–2002 (33, 37). However, in many health care facilities, HALD cases have been discovered after the implementation of *Legionella* environmental monitoring and clinical surveillance (7, 32). Our results indicate the presence of disease-causing *L. pneumophila* in the hospital environment; therefore, warrant the necessity of investigating *Legionella* among all patients having nosocomial pneumonia in this facility.

### *L. pneumophila* Sequence-Based Typing

Over the last decade, SBT analysis has been accepted as a gold standard for the genotyping of *L. pneumophila* isolates. Besides, SBT can be applied to study the genetic diversity and clonal expansion of *L. pneumophila* populations. The method has the advantage of better classification potential, good reproducibility, and is more economical (4, 16–18). Our study represents SBT analysis of environmental *L. pneumophila* isolated from the water systems of a tertiary health care center in India, and the results depict the genetic diversity of this pathogen even though all isolates were derived from the human-made environment. The IOD of the 44 environmental isolates was found to be 0.929 that is higher compared to studies reported from Canada (IOD, 0.888), Japan (IOD, 0.886), and the USA (IOD, 0.751), but lower than those reported from Singapore (IOD, 0.970) (4, 25, 38, 39). We also found that non-Lp1 isolates (IOD, 0.955) are more variable than Lp1 isolates (IOD, 0.824), which could be due to the high prevalence of ST1 among the Lp1 isolates. Additionally, NPW isolates were found to have high IOD (IOD, 0.933) followed by PW (IOD, 0.920) and CT isolates (IOD, 0.888).

ST1, the most common ST distributed throughout the world, was the dominant profile in this study (4, 6, 25, 26, 39). Multiple outbreaks due to ST1 strains have been reported from the USA, Canada, and Europe (4, 39). ST1 isolates are well-adapted to survive in human-made water environments such as a cooling tower, and the ability of this ST to adapt to natural water environments, including hot springs and soil, is found to be low (25). From studies conducted in Japan and South Korea, it was observed that most of the Lp1 environmental isolates, especially those from CT, belonged to ST1 (25, 26). In contrast to these findings, in the present study, ST1 was the most dominant ST in Lp1 isolates from PW (42.8%), followed by those from NPW (33.3%) and CT (16.6%). In a Chinese study, ST1 accounted for 92.3 and 53.1% of the isolates from PW and CT, respectively (6).

Additionally, in a US study, it was reported that ST1 was the dominant ST in both PW and CT isolates (4). These differences could be possibly due to the predominance of specific STs or unique strains in various water systems types in these countries. Besides, the genes coding for proteins (e.g., flagellum, pilin, outer membrane protein, macrophage infectivity potentiator, Zinc metalloproteinase) may interact with external environments; therefore, an isolate to get adapt to an environmental source may have a particular ST suitable for each environment (40).

*L. pneumophila* serogroup 1 STs previously reported from Japan, China, and South Korea (countries belong to East Asia) were compared with STs obtained in this study. The results indicated that some STs (e.g., ST1) are distributed world-wide across different countries, but some clones and STs could be unique circulating only in specific regions. The possibility of recombination events between *L. pneumophila* STs has been previously reported (6, 25). ST161 (11,4,3,1,1,1,1) in CC-A was reported to be a recombinant between ST1 (1,4,3,1,1,1,1) and ST154 (11,14,16,16,15,13,2) the determined primary founders of CC-A and CC-B, respectively (Figure 3). Similarly ST2850 (2,10,3,28,19,4,3) which is identified in our study was a recombinant of ST1464 (2,10,15,28,19,4,3) and ST1 (1,4,3,1,1,1,1) the predicted primary founders of CC-1 and CC-2, respectively (Figure 2).

Querying the ESGLI SBT database (available at http://www.esgli.org), it was found that of the 23 STs we obtained in this study, 11 were new to the database, and among the remaining STs, 3 (ST1095, ST1464, ST2210) were unique to Asia. Notably, all three STs were reported to be isolated from environmental sources, and no clinical infections due to these STs have been documented so far (Table 5). It will be interesting to see whether these STs will be associated with any LD cases or outbreaks in the future. The goeBURST analysis has shown that of the 11 STs that are newly identified in this study, two STs have single-locus variants (SLVs), 5 STs have double-locus variants (DLVs), and 2 had triple-locus variants (TLV) within our STs. Besides, querying the ESGLI database, it was found that 7/11 STs are having SLVs abroad (Table 5). Further studies are needed to determine if these STs will persist in this geographical region or expand to other continents.

**Table 5:** The ESGLI database information regarding the *L. pneumophila* STs identified in this study (as of 11, January, 2020).

**S.no**	**Sequence type**	**Distribution of STs**		**Reported geographic regions/countries (according to ESGLI SBT database)**	**Comments**
		**Environmental isolates**	**Clinical isolates**		
1	ST1	yes	yes	Europe, North and Central America, Australia, Asia, Africa	Most common ST reported world-wide, community-acquired, nosocomial, and travel-associated infections are reported
2	ST48	yes	yes	Europe, Asia, North America	Community-acquired, nosocomial, and travel-associated infections are reported
3	ST93	yes	yes	Europe, Asia	Community-acquired, nosocomial, and travel-associated infections are reported
4	ST114	yes	yes	Asia, Europe, North America	Community-acquired and nosocomial infections are reported
5	ST134	yes	yes	Asia, Europe, North America	Travel associated and community-acquired infections are reported
6	ST322	yes	–	Russia, India^*^	Reported from environmental sources, infection due to this ST has not been reported
7	ST763	yes	yes	Europe, Canada, Asia, USA	Community-acquired pneumonia due to this ST has been reported
8	ST1095	yes	–	Asia (Indonesia, Japan, China, India^*^)	Only isolated from environmental sources, clinical infection due to this ST has not been reported
9	ST1376	yes	yes	North and Central Europe, India^*^	Community-acquired pneumonia due to this ST has been reported
10	ST1417	yes	–	Europe (Switzerland), Asia (China, India^*^)	Reported from water systems during a few instances, clinical infections are not documented
11	ST1464	yes	–	Asia (Indonesia, China, India^*^)	Reported from water systems during a few instances, clinical cases are not yet reported
12	ST2210	yes	–	Asia (Cambodia, India^*^)	Reported from environmental sources, clinical infections due to this ST are not documented
13	ST2848	yes	–	India^*^	First time reported in the present study, closely related to ST2775
14	ST2849	yes	–	India^*^	First time reported in the present study, closely related to ST7, ST1011, and ST560
15	ST2850	yes	–	India^*^	First time reported in the present study, closely related to ST728, ST1609, ST1464, ST1297, and ST2180
16	ST2854	yes	–	India^*^	First time reported in the present study
17	ST2855	yes	–	India^*^	First time reported in the present study
18	ST2865	yes	–	India^*^	First time reported in the present study
19	ST2866	yes	–	India^*^	First time reported in the present study, closely related to ST1356
20	ST2867	yes	–	India^*^	First time reported in the present study, closely related to ST1464
21	ST2868	yes	–	India^*^	First time reported in the present study, closely related to ST187, ST31, ST468, ST2876, and ST2048
22	ST2869	yes	–	India^*^	First time reported in the present study
23	ST2874	yes	–	India^*^	First time reported in the present study, closely related to ST242, ST1017, and ST2846

* *present study*.

*ESGLI, European study group for Legionella infections; SBT, Sequence-based typing; ST, sequence type*.

According to the ESGLI SBT database, few STs described in this study are found to be associated with LD cases and outbreaks. Clinical infections due to STs, including ST1, ST48, ST93, ST114, ST134, ST763, and ST1376, are reported in the ESGLI SBT database (Table 5). These STs that are common to clinical isolates may have an increased ability to cause LD; therefore, their water system sources represent a potential source of legionellosis. ST1 and ST134 (representing CC-2 in this study, Figure 2) were associated with multiple sporadic cases and outbreaks in many parts of the world (4, 39). Furthermore, it was hypothesized that similar to certain *Legionella* spp. and serogroups, few STs also have an enhanced ability to cause infections in humans (41). Hence *Legionella* control strategies can specifically target these STs that cause the majority of human infections.

During the study period, we could not obtain clinical isolates from patients for genotyping, but SBT was performed directly on the respiratory sample (BAL fluid) of an LD case-patient who was diagnosed by PCR and urine antigen testing (BinaxNOW, Alere, USA). Briefly, we used a nested PCR derived SBT directly to the DNA isolated from respiratory fluid and assigned allele, and finally, a sequence type (ST) using the online ESGLI SBT database (42). We identified that the infection was due to ST1, but was not able to perform epidemiological investigations to determine the possible source of the infection as the patient has acquired infection from another facility. Further studies are needed to analyze the correlation between *L. pneumophila* environmental and clinical isolates from this region.

Even though we described the distribution and classification of environmental *L. pneumophila* isolates in a healthcare facility, in future, large-scale studies using whole-genome sequencing (WGS) are needed to classify *L. pneumophila* environmental and clinical isolates and to identify factors that give fitness to this bacteria to survive in the aquatic environments and to infect humans.

## Data Availability Statement

The original contributions presented in the study are publicly available. This data can be found here: GenBank MT890971.

## Ethics Statement

The studies involving human participants were reviewed and approved by The ethics committee of AIIMS, New Delhi. The patients/participants provided their written informed consent to participate in this study.

## Author Contributions

RC: conceptualization, project administration, resources, validation, writing-review, and editing. KS and RC: data curation. KS, RC, and BT: formal analysis. KS and RC: investigation and methodology. KS and EV: software. RC, SK, AD, RG: supervision. KS: writing-original draft. All authors contributed to the article and approved the submitted version.

## Conflict of Interest

The authors declare that the research was conducted in the absence of any commercial or financial relationships that could be construed as a potential conflict of interest.
